# Dental Education in an Era of COVID-19: Kuwait’s Experience

**DOI:** 10.3390/ijerph18115606

**Published:** 2021-05-24

**Authors:** Jagan Kumar Baskaradoss, Adel Al-Asfour

**Affiliations:** 1 Department of Developmental and Preventive Sciences, Faculty of Dentistry, Kuwait University, P.O. Box 24923, Safat 13110, Kuwait; 2 Department of Surgical Sciences, Faculty of Dentistry, Kuwait University, P.O. Box 24923, Safat 13110, Kuwait; adel.alasfour@ku.edu.kw

**Keywords:** blended-learning, COVID-19, dentistry, education, e-learning

## Abstract

The coronavirus disease 2019 (COVID-19) pandemic has had a significant impact on dental education worldwide. Due to the rapid spread of COVID-19 across Kuwait, it was essential for the Faculty of Dentistry (FoD) at Kuwait University to make appropriate modifications to the functioning of the dental school. The FoD’s goal was not only to ensure a safe environment for its staff, students, and patients but also to sustain the students’ academic progression. The FoD adopted several measures including the establishment of a COVID-19 response team, adoption of a blended-learning model, and phase-wise re-opening of the dental center. This paper discusses on the strategies that the FoD adopted, in response to the challenges posed by the pandemic.

## 1. Background

Globally, over 165 million cases of coronavirus disease 2019 (COVID-19), including more than 3.4 million deaths, have been reported as of 20 May 2021 [1]. The pandemic, caused by the severe acute respiratory syndrome coronavirus 2 (SARS-CoV-2), has rapidly overwhelmed the healthcare system in most countries. As of 20 May 2021, the Ministry of Health (MoH) in Kuwait had reported more than 294,000 confirmed COVID-19 cases, including 1703 deaths, with a recovery rate of 95.3% and a fatality rate of 0.6% [2]. The first reported case of COVID-19 in Kuwait occurred in the last week of February 2020 [3]. Soon after, an exponential increase in the number of COVID-19 cases in the country was observed.

As COVID-19 is predominantly transmitted through respiratory droplet/contact, the dental community was especially concerned. It was believed that the transmission potential of the virus might be higher inside the dental office, as many dental procedures generate droplets (5–12 μm) and aerosols (<5 μm) that may contain viral particles from infected individuals [4]. The nature of the dental setting puts both the dentist/dental team and the patient at a high risk of cross-infection. In the US, the Occupational Safety and Health Administration (OSHA) classified dentists in the ‘very-high-risk’ category [5]. In the early stages of the pandemic, the School and Hospital of Stomatology at Wuhan University reported nine confirmed cases of COVID-19 (including three doctors, three nurses, two administrative staff, and one postgraduate student) [6]. It was strongly recommended that dental practices and hospitals follow strict and effective infection control protocols [7]. Except for dental emergency procedures, most dental schools in the United States had suspended all clinical activities [8]. The Association of Dental Education in Europe (ADEE) also reported a similar response among the European dental schools [9].

Following the recommendations of the various international public health agencies, such as the World Health Organization (WHO) and the Centers for Disease Control and Prevention (CDC), the Faculty of Dentistry (FoD) at Kuwait University initiated several measures to mitigate the spread of COVID-19. The primary focus was to ensure a continuity of dental education in a safe environment by adhering to the recommendations of the health departments. This paper describes the experiences and the protocols that was adopted by the FoD since the beginning of the pandemic.

### 1.1. FoD’s Experience

The Faculty of Dentistry follows a seven-year dental curriculum (four years of pre-professional/pre-clinical courses conducted jointly with the Faculty of Medicine and three years of clinical training at the FoD) and accepts an average of 28 students each year. There are 65 teaching faculty members and approximately 80 administrative assistants. The clinical training is provided at the Kuwait University Dental Center (KUDC), which has 105 dental assistants and technicians. From March to August 2020, the FoD was closed, as per the instructions of the Ministry of Higher Education in Kuwait. During this period, many faculty members and students assisted the Ministry of Health’s (MoH’s) COVID-19 response by volunteering at COVID-19 testing centers or by joining the MoH’s contact tracing team. This period was utilized to train the staff and the students on the use of various learning management systems (LMSs). The online training on the use of different LMSs was undertaken by the information technology (IT) department of Kuwait University. The training was provided for the Microsoft (MS)-Teams, Moodle, and Blackboard LMSs. Following this, a survey was undertaken to obtain feedback from the staff and students on their preferred LMS. For providing lectures, MS Teams was favored by the respondents for its ability to promote interaction between the students and the instructor. For assessment purposes, Moodle was preferred by both faculty and students for its user-friendly interface.

From August 2020, the FoD adopted a blended-learning model, which combined e-learning platforms for the lectures and traditional on-site model for the clinical training.

### 1.2. Lectures

MS Teams provided a reliable interactive platform for delivering live lectures. An asynchronous option was also provided to the students where faculty members could upload pre-recorded lectures to MS-Teams. Most of the lectures were recorded and uploaded to the video application MS-Stream. Links to these recorded lectures were posted in the interactive discussion panel in MS-Teams, where the students could access them at their convenience. Online examinations were conducted using the Moodle Quiz feature of the Moodle LMS, which provided a well-controlled environment. Additionally, the students were instructed to turn on the camera of their mobiles showing the computer screen to help with the invigilation during the online exams. 

### 1.3. Simulation Labs

Simulation labs are one of the safest methods for students to acquire practical clinical skills in dentistry [10]. The FoD began simulation laboratory courses in August 2020, adhering to the social distancing policy of the FoD. It was decided that students would be divided into small groups to ensure that the required distance between students (2 m) could be maintained. Additionally, the students were separated by transparent plastic boards. Although the start date of the simulation labs was delayed, after adopting the necessary steps, the labs functioned successfully throughout the rest of the semester.

### 1.4. Clinical Training

Several training sessions addressing key areas, such as infection control protocols, the proper use of personal protective equipment (PPE) and hand hygiene, were organized for the staff members, and the students. The staff working in all of the different departments at FoD, were provided training on the COVID-19 protocols.

The recommendations of the American Dental Association [11] were adopted for determining the risk level of various dental procedures. Workstations (e.g., clinics, dispensaries, dental stores, reception, offices, laboratories, sterilization areas), in addition to other areas that may lead to close contact (defined as less than two meters) among employees and patients (e.g., waiting rooms, faculty/student/nurse lounges, locker rooms, check-in areas, and a pathway of entry and exit), were modified to adhere with the social distancing guidelines. Visual cues were used (e.g., floor markings and signages) to encourage physical distancing and all of the seating within the premises were organized accordingly. Patients were allowed to enter the clinic only at their scheduled appointment time.

### 1.5. Re-Opening the Dental Center

**Figure 1:**
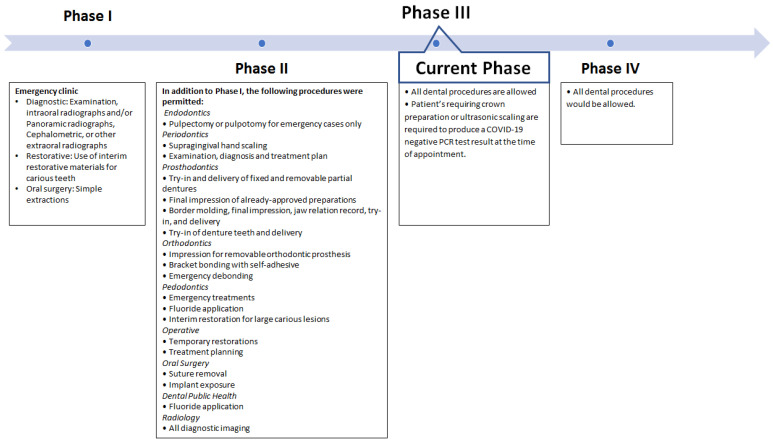
Dental clinic re-opening phases and the clinical procedures permitted in each phase.

It was decided that the dental clinic would reopen in four phases. A list of the clinical procedures that were permitted in each phase is shown in Figure 1.

### 1.6. Preparatory Phase

This phase lasted for two months, from June to July 2020. This phase involved the following: (1) a re-evaluation of the available stocks of PPE, (2) infection control training for dental assistants/clinical staff and students, (3) re-training for the housekeeping staff and cleaners in disinfection protocols and safe work practices, (4) the maintenance of dental chairs and the sterilization room by contractors, and (5) an operational assessment of the ventilation and water systems.

### 1.7. PHASE I

From August 2020, for a period of four weeks, only emergency dental care was provided at the dental center. Students were only permitted to review the treatment plans of their ongoing cases. 

### 1.8. PHASE II

From September to October 2020, students were permitted to perform most of the non-aerosol generating procedures. Procedures involving the use of a handpiece were not allowed, except for an emergency pulpectomy or pulpotomy.

### 1.9. PHASE III

Since October 2020, the clinic’s functioning has mostly returned to that of the pre-COVID-19 period. Students can perform all dental procedures, with only selected procedures (ultrasonic scaling and prosthodontic crown preparation) requiring the patient to produce a negative polymerase chain reaction (PCR) certificate. Weekly meetings are held at the FoD to assess the clinical situation and to determine the safety of continuing clinical work.

### 1.10. PHASE IV

This phase is scheduled to begin post-pandemic, when the clinic’s functioning would return to the pre-COVID-19 period. 

## 2. Contact Tracing Team and Its Efforts

**Figure 2:**
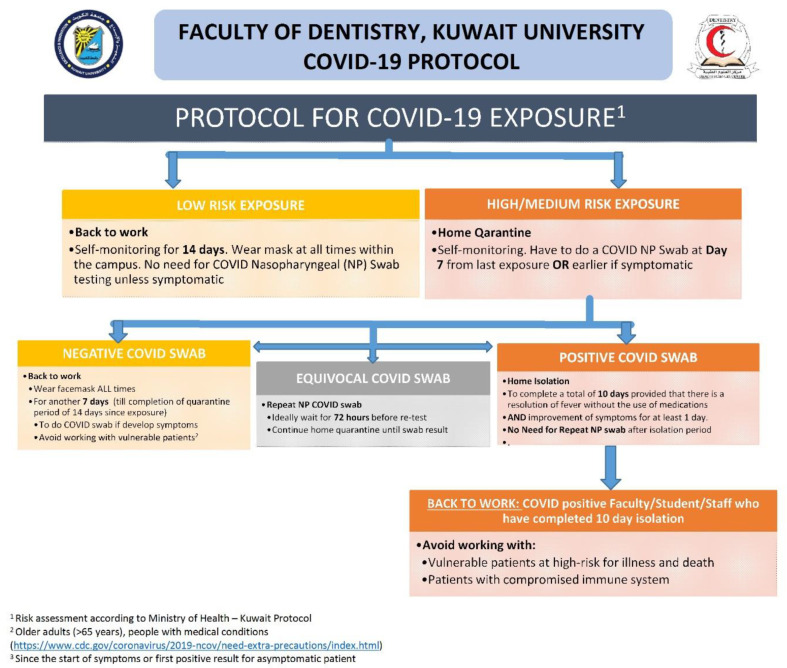
Protocol for COVID-19 exposure among the staff members, and the students adopted by the Faculty of Dentistry, Kuwait University.

The FoD established a COVID-19 response team comprising of public health specialists and representatives from each of the clinical disciplines. One of the main objectives of this team was to act as the contact point for the staff and students to report on any exposure they might have had with a COVID-19-positive patient. When contacted, the team would assess the risk of exposure for the staff or student and provide them with the necessary recommendations. The team adopted the protocol of Kuwait’s MoH for assessing the risk of exposure (Figure 2).

### 2.1. Protocol

Any staff member or student who came in close contact with a known COVID-19 positive patient, or who has flu-like symptoms (i.e., a sore throat, fever, cough, or shortness of breath), or were tested positive for COVID-19, were asked to immediately notify the COVID-19 response team and to quarantine themselves. They would then be contacted by a public health specialist from the FoD to assess the risk of exposure and provide suitable recommendations. The following data were collected from every individual using a pre-designed form: (1) demographic characteristics, (2) date of onset of symptoms, (3) date of testing and test result, (4) period of isolation or quarantine, and (5) details of contacts. Close contacts were advised to quarantine at home for 14 days after their last exposure to a positive COVID-19 case. All contacts were advised to go for testing and the details of the nearest COVID-19 testing center were provided. The entire process was conducted in a time-bound manner.

### 2.2. Results of Contact-Tracing

As of 20 May 2021, eight academic staff, nine dental assistants, and nine students had been tested positive for COVID-19. The probable source of infection was not identified for most of the individuals and the information obtained through contact tracing indicated community transmission. Through contact tracing, it was determined that none of the interactions that the COVID-19 positive individuals had with other members of the faculty within the campus could be categorized as a “close contact”. A “close contact” was defined as anyone who had spent more than 15 min in direct face-to-face contact within two meters of a confirmed case, lived in the same household or shared any leisure or professional activity in close proximity with a confirmed case without appropriate PPE in any setting, within the period from two days before symptom onset (or, for asymptomatic patients, two days prior to a positive specimen collection) until the time that the confirmed case was isolated [3]. As of 20 May 2021, six academic staff, seventeen dental assistants, and nine students had reported to have had a “close contact” with a confirmed COVID-19 positive patient outside of the university campus. The transmission of disease between colleagues within the faculty was possibly prevented by the mandatory mask-wearing policy and by the strict adherence to the social distancing policies of the FoD.

## 3. Discussion

With the pandemic growing at an alarming rate in Kuwait, the primary responsibility of the FoD was to provide a safe and robust learning environment for its students. The FoD adopted a blended-learning model for the undergraduate teaching. 

The planning and preparation for the change from a traditional classroom teaching model to an e-learning platform was well accepted by the staff and students alike. Several studies have reported a similar experience regarding the effectiveness and acceptability of e-learning in dental education [12,13]. A recent study reported that dental students were comfortable with technological adaptations for didactic curriculum and are in favor of wearing masks, social distancing, and a liberal use of sanitizers within the campus [14].

In the US, the Centers for Disease Control and Prevention (CDC) published guidelines for reopening institutions of higher education based on the five risk levels associated with educational activity. The categories range from “lowest risk” (which comprises of activities that are limited to virtual learning only) to “highest risk” (e.g., individuals are not spread apart and materials and supplies are shared) [15]. The traditional methods of teaching in any dental school would constitute “highest risk”. Adopting a blended-learning model reduced the risk of COVID-19 transmission. Utilizing the e-learning platforms helps to avoid any unnecessary aggregations of people and the associated risk of infection [6]. Hung et al. [14] reported over half of dental students worried about contracting COVID-19 from providing patient care or interacting with others at school during the COVID-19 outbreak.

According to the CDC, “There is neither expert consensus, nor sufficient supporting data, to create a definitive and comprehensive list of aerosols generating procedures for dental healthcare settings. Commonly used dental equipment known to create aerosols and airborne contamination include ultrasonic scaler, high-speed dental handpiece, air/water syringe, air polishing, and air abrasion”. An earlier study conducted by Iyer et al. [8], discusses strategies to deliver emergency care during the pandemic, such as patient screening, remote consultations, and the appropriate use of PPE. Fortunately, the infection levels among dental personnel appear to be relatively low, probably because of the use of PPE, high volume suction, etc. A nationally representative study on the COVID-19 infection rates among US dentists reported that less than one percent of dentists were found to be COVID-19 positive [16]. This could be due to the precautions taken by the various dental institutions ensuring social distancing and use of recommended PPE.

The FoD was successful not only in ensuring a safe environment for its staff, students, and patients but also in sustaining the students’ academic progression. All three batches of dental students were able to complete their didactic and clinical requirements within the scheduled time period. One of the advantages the FoD had in implementing the new system was the relatively small class size. This enabled an easy transition from the traditional face-to-face teaching system to a blended-learning model. Examinations were conducted entirely online, and the evaluation of the required clinical competencies were conducted as planned. Taking into consideration the restrictions due to the pandemic, some of the clinical requirements were reduced minimally (<10%) for the graduating batch of students. 

The dental center started functioning in phases. This provided the faculty and students with adequate time to adapt to the changes implemented due to COVID-19. The decision to move from one phase to another was taken only after carefully assessing all of the relevant factors, such as faculty/student experience, staff preparedness, and the MoH’s recommendations. To meet the challenges presented by the pandemic, it is important for every dental school to follow the local health department’s recommendations to ensure a safe environment for patients, students, and faculty [17]. As recommended by various international health agencies, the FoD ensured that students and faculty wore suitable PPE’s, every patient’s temperature and relevant medical history were recorded and high-power suction along with rubber dam isolation were routinely used for all patient’s [18,19,20]. Most non-emergency dental services, especially those generating aerosols, were undertaken only in the third phase. As recommended by Meng et al. [6], rubber dams and high-volume ejectors were employed in dental clinics to minimize the aerosol production during dental treatments. Similar to reports from other countries, the FoD decided to divide the students into smaller groups to minimize interactions, thereby, minimizing the risk of the three C’s: closed indoor venue, crowded place and close contact [14].

One of the key initiatives undertaken by the FoD during this pandemic was the establishment of a COVID-19 response team. This helped in providing timely recommendations to the staff and students who had either contracted the disease or had a high-risk exposure to a COVID-19 case. There were no instances of disease transmission occurring within the FoD’s premises. However, given that the community transmission of COVID-19 is high across the country, staff and students might still acquire COVID-19.

## 4. Conclusions

Establishing a COVID-19 response team, adopting a blended-learning model and introducing a phase-wise re-opening of the dental services under strict monitoring proved to be very effective measures to ensure a safe learning environment at the FoD. The COVID-19 pandemic has had a significant impact on dental education and with the uncertainties regarding the future trends of the pandemic, it is of paramount importance for every organization, including dental schools, to adhere to the recommendations provided by local health departments.
